# IgA deficiency reveals a microbiota-dependent pathway to gluten sensitivity

**DOI:** 10.1080/19490976.2026.2724175

**Published:** 2026-09-18

**Authors:** Ryan A. W. Ball, Ahmed D. Mohammed, Amy Jolly, Kenny Johnson, Summer Sklenicka, Melana Kucherina, Tori Peacock, Evan Liu, Kristen M. Hogan, Maredith Baird, Maria Marjorette O. Peña, Prakash Nagarkatti, Mitzi Nagarkatti, Jason L. Kubinak

**Affiliations:** a Department of Pathology, Microbiology, and Immunology, University of South Carolina School of Medicine, Columbia, SC, USA; b Department of Biological Sciences, University of South Carolina, Columbia, SC, USA

**Keywords:** Immunology, microbiome, gluten sensitivity, streptococcus, IgA deficiency, mucosal immunology, small intestine

## Abstract

Selective IgA deficiency (sIgAD) is the most common primary immunodeficiency and increases susceptibility to gluten-related enteropathies, but the underlying mechanisms of pathogenesis are unknown. Utilizing IgA^−/−^ mice and wild-type controls, we investigated how dietary gluten shapes susceptibility to small intestinal inflammation. We found that IgA^−/−^ mice developed gluten-induced villus blunting in the ileum and enhanced T_h_17 responses. Exposure to a gluten-free diet prevented villus blunting in IgA^−/−^ mice. Dietary gluten promoted the expansion of *Streptococcus* and *Desulfovibrio* species, depletion of members of the order *Lactobacillales*, and shifts in microbial metabolic pathways related to lipid metabolism. Next, to determine if gluten sensitivity is microbiota-dependent, germ-free colonization experiments were performed using complete microbiota transfers or mono-colonization with a single *Streptococcus* species (*Streptococcus lutetiensis*), both of which were sufficient to recapitulate gluten-sensitive enteropathy. Our findings demonstrate that dietary gluten promotes small intestinal inflammation and mucosal remodeling in IgA-deficient mice through microbiota-dependent mechanisms. This work highlights a key role for sIgA in maintaining immune homeostasis at the diet–microbiota interface and reveals a novel microbial pathway underlying gluten sensitivity.

## Introduction

The intestinal mucosa is home to a complex and dynamic microbial ecosystem comprising hundreds of bacterial species that interact with the host's immune system to maintain homeostasis and prevent disease.^1^ Among the key regulators of this host-commensal relationship is secretory IgA (sIgA), the most abundant antibody in the mucosal environment.^2^ By targeting and neutralizing pathogens while promoting microbial diversity, sIgA shapes the taxonomic composition, spatial organization, and metabolic activity of the gut microbiota.^3^ Selective IgA deficiency (SIgAD) is the most common primary immunodeficiency, with reported prevalence varying widely by ethnicity, population, and diagnostic criteria. Estimates range from approximately 1:143 to 1:1,000 in many Western and Middle Eastern populations, with lower reported frequencies in several Asian populations.^4^ SIgAD is associated with an increased risk of inflammatory conditions, particularly within the gastrointestinal tract.^5^ Notably, patients with sIgAD are 10–15 times more likely to develop celiac disease (CeD), an enteropathy triggered by exposure to dietary gluten.^6^ Despite this association, the mechanisms linking sIgAD to gluten sensitivity remain poorly understood.

Gluten is a complex protein found in wheat, barley, and rye that provides the elastic properties desirable in many food products.^7^ However, these same properties contribute to gluten sensitivity, a term encompassing a range of pathological responses to the ingestion of dietary gluten, including CeD and non-celiac gluten sensitivity (NCGS).^8^ Gluten sensitivities can manifest with gastrointestinal symptoms such as diarrhea, abdominal pain, and bloating, as well as systemic effects like anemia, brain fog, and ataxia.^9^ ^,^ ^10^ In severe cases, chronic inflammation can lead to small intestine enteropathy (SI enteropathy), resulting in long-term damage to the intestinal lining, malabsorption of nutrients, and related complications.^11^ Although CeD is often driven by genetic predisposition (HLA-DQ2/DQ8 alleles), most individuals who carry these genetic risk factors do not develop the disease, suggesting that additional environmental factors, particularly the gut microbiota, contribute to disease onset and progression.^12-16^

The role of the gut microbiota in gluten sensitivity is poorly understood, but evidence suggests two possible mechanisms: (1) the enhanced production of immunogenic gluten peptides or gluten-mimicking peptides by commensal bacteria,^17^ ^,^ ^18^ and/or (2) a prebiotic effect of gluten that results in the outgrowth of inflammatory pathobionts.^14^ ^,^ ^19^ While most studies have focused on the enzymatic processing of gluten by gut microbes, the potential for dietary gluten to reshape both the composition and metabolic function of the microbiota—particularly under conditions of impaired immune regulation—has received far less attention. Given the central role of secretory IgA in modulating both microbial community structure and functional capacity, its absence may permit the expansion of proinflammatory taxa and the emergence of pathogenic microbial activities in response to dietary gluten, thereby enhancing disease susceptibility in prone individuals.

Previously, our group demonstrated that B-cell-insufficient CD19^−/−^ mice^20^ and B-cell-deficient J_H_−/−^21^ mice develop gluten-induced dysbiosis leading to SI enteropathy. These studies suggest that impaired humoral immunity predisposes the host to gluten-induced intestinal damage through microbiota-dependent mechanisms. Here, we extend these findings to a model of sIgAD and reveal that the absence of sIgA alone is sufficient to drive gluten-dependent immune activation and epithelial remodeling in the small intestine. We show that dietary gluten promotes the outgrowth of specific microbial taxa—most notably *Desulfovibrio spp.* and *Streptococcus lutetiensis*—and that colonization of GF mice with *S. lutetiensis* is sufficient to induce inflammatory T-cell expansion and SI enteropathy in a gluten-dependent manner. These findings reveal that dietary gluten can disrupt the host–microbe interface, and in the absence of sIgA-mediated regulation, this disruption is sufficient to drive mucosal inflammation and tissue damage. Together, our results identify a mechanism by which sIgAD amplifies host susceptibility to microbiota-driven intestinal pathology and highlight the importance of humoral immunity in maintaining tolerance to microbial and dietary antigens.

## Materials and methods

### Experimental animals

A long-term breeding colony of IgA^−/−^ mice (originally purchased from the Mutant Mouse Resource and Research Center (MMRRC)) has been maintained by the Kubinak Lab at the University of South Carolina. All mice are housed under identical SPF conditions in one environmentally controlled room (70°F, 50% relative humidity, 12:12 light:dark cycles) and given *ad libitum* access to autoclaved drinking water and a standardized irradiated mouse chow (Envigo 2920X). Male and female mice were used in all experiments. Experimental data were derived from eight-to-ten-week-old pure-bred WT and IgA^−/−^ mice.

For gnotobiotic colonization experiments, 8–10-week-old C57BL/6 germ-free (GF) mice were used. All animal use strictly adhered to federal regulations as well as the guidelines set forth by the University of South Carolina Institutional Animal Care and Use Committee (IACUC) (Protocol#101182). GF C57BL/6 mice were obtained from a breeding colony maintained at the University of South Carolina (USC) Mouse Experimentation and Gnotobiotic Core Facility (MEGCF). The mice were procured from the National Gnotobiotic Rodent Resource Center of the University of North Carolina at Chapel Hill and were descendants of the original colony derived from the Jackson Labs C57BL/6J embryos. GF animal care procedures and use were all in accordance with federal regulations and approved by the USC IACUC (protocol #2779-102068-091225). GF mice were maintained in sterile flexible film isolators (Class Biologically Clean, Madison, WI), housed in open-top cages with autoclaved bedding, and kept on a 12-hour light:dark cycle under controlled conditions (temperature: 25 ± 2°C, humidity: 45%–60%) with *ad libitum* access to sterilized water and food. GF mice were fed 2019 Teklad Global 19% protein extruded diet (Cat. No. 2019S) that was gamma-irradiated and autoclaved to ensure complete elimination of microorganisms. The diet is formulated to retain adequate nutrients for rodents after autoclaving. To ensure that mice were microbiologically clean prior to use in experiments, germ-free status was checked weekly by culturing fresh fecal pellets from each isolator. For anaerobic bacterial culture, Brucella agar plates containing 5% sheep blood, hemin, and vitamin K (BD BBL) were pre-reduced prior to inoculation and incubation in an anaerobic chamber. Aerobic cultures were plated on Trypticase Soy Agar plates containing 5% sheep blood (BD BBL). All cultures were incubated at 37°C for 2–3 d, followed by 5–7 d at room temp. In addition, 16S rRNA PCR was performed using DNA extracted from pooled fecal samples collected from each isolator monthly. For negative controls, sterile PBS or water was used, and for positive controls, feces collected from SPF mice were used.

### Histological analysis of SI enteropathy

**Figure 1.**
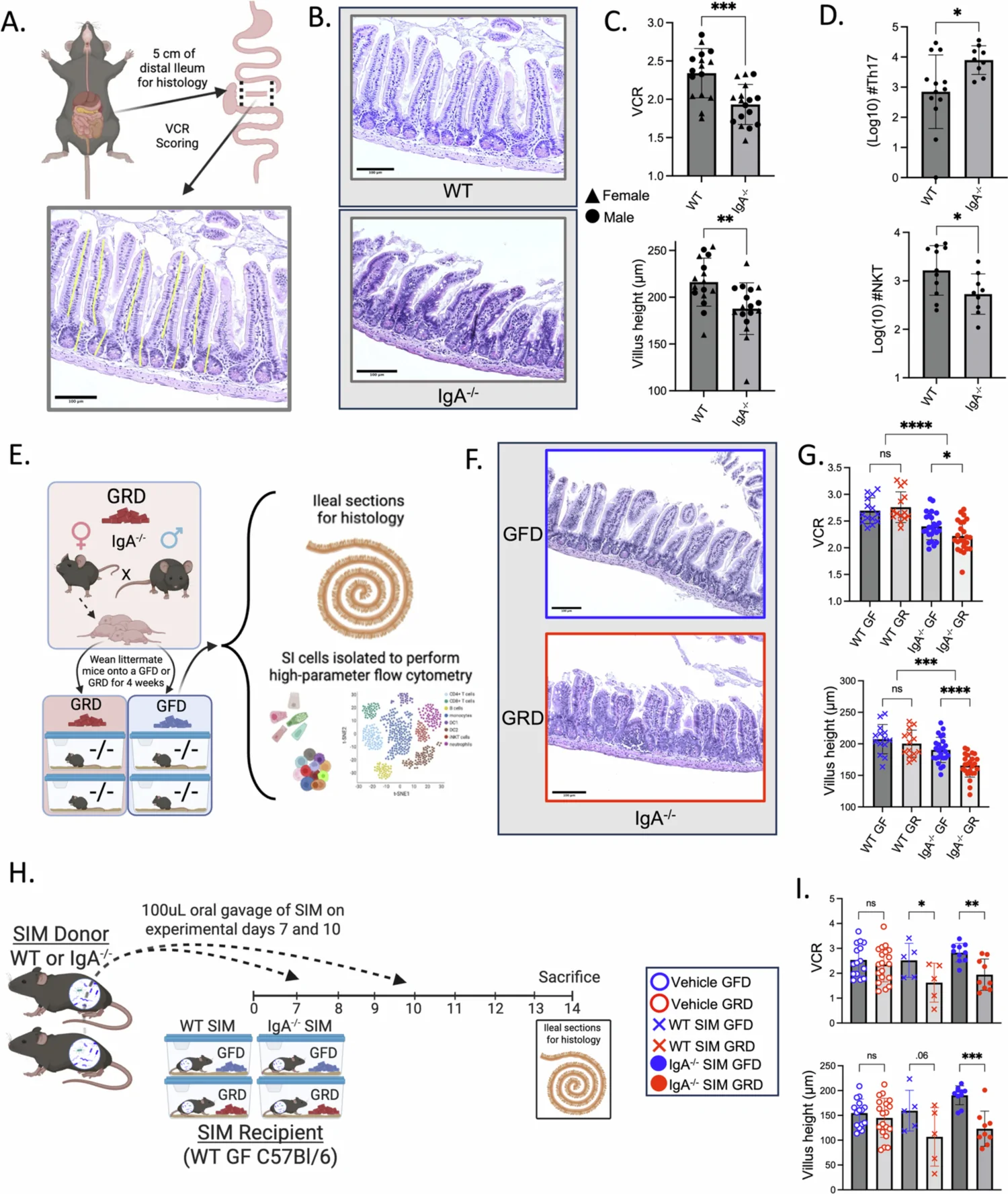
IgA-deficient mice develop gluten sensitivity which is microbiota-dependent. (A) A schematic of ileum sampling and VCR scoring is shown. (B) Representative images of IgA^−/−^ and WT mice are shown. (C) VCR and villus height for conventionally housed WT (*n* = 16) and IgA (*n* = 16) mice on a standard gluten-containing mouse chow. (D) Absolute abundance of Th17 cells (TCR β+ CD4+ CD69+ IL17+) and NKT cells (CD3+ NK1.1+ TCR β +)I *n* conventionally housed littermate WT (IgA^+/^^−^) (*n* = 12) and IgA^−/−^ (*n* = 9) mice. (E) Schematic of gluten experiments. (F) Representative images of IgA-deficient mice singly housed on a GFD or GRD. (G) VCR scoring and villus height for singly housed WT (GFD *n* = 14 and GRD *n* = 14) and IgA^−/^^−^ (GFD *n* = 25 GRD *n* = 24) mice on a GFD or GRD (pooled three replicate experiments). (H) Schematic of germ-free WT and IgA SIM colonization gluten experiment (I) VCR scoring and villus height for singly housed WT(*n* = 10) or IgA^−/^^−^ (*n* = 19) SIM colonized WT/BL6 germ-free mice on a GFD or GRD (pooled two replicate experiments). Two-way ANOVA showing “genotype”, “diet” or “genotype × diet” or “colonization”, “diet” or “colonization × diet” interactions with ad hoc comparisons to show significant interactions; ns = not significant, * = *p* < 0.05, *** = *p* < 0.001, **** = *p* < 0.0001.

In total, 5 cm of ileum (proximal to cecum) was removed from mice, cleaned, and placed into 10% formalin for 24–48 hours. Tissue was submitted for processing to the USC Instrumentation Resource Facility Histology Core. The tissue was embedded with paraffin, sectioned, and H&E stained. Ileal sections were imaged under an EVOS microscope at 20× magnification. A minimum of 6 areas of 5 consecutive villi and adjacent crypts were manually selected based on the clarity of the section. Villi and crypt ratios were calculated by measuring the villus height (Vh) and adjacent crypt depth (Cd) on ImageJ and then dividing Vh by Cd to obtain a final ratio. An average of the five Vh and five adjacent Cd were measured per area and then an average of the 6–10 areas was calculated per mouse for statistical analysis (Figure 1A).

### Dietary gluten manipulation experiments

For gluten experiments, animals were maintained on either a gluten-free diet (GFD) (Bioserv; diet#S3156- AIN-93G;) or a nutritionally matched gluten-rich diet (GRD) that incorporated gluten (rather than casein) as the dominant protein source (Bioserv; diet# S7784- AIN-93G,-Modified Wheat Gluten). For these experiments, mice were weaned onto their respective diets at three weeks of age and singly housed for four weeks with *ad libitum* access to diets. After 4 weeks, the mice were sacrificed, and materials were collected for histology, flow cytometry, and 16S rRNA sequencing.

### Germfree (GF) small intestinal microbiota (SIM) colonization experiments

C57BL/6 GF mice maintained at the University of South Carolina Gnotobiotic Facility were singly housed in individually ventilated Allentown cages at the USC MEGCF and placed on either a nutritionally matched GFD or GRD for one week prior to colonization. Small intestine luminal contents were collected from conventionally housed WT or IgA^−/−^ donor mice eating a standard gluten-containing chow diet and resuspended in sterile PBS. Coarse particulate material was removed by low-speed centrifugation at 50 × g, and the resulting supernatant was used as the bacterial suspension. GF recipient mice were orally gavaged with 100 μL of either WT or IgA^−/−^ SIM suspension on day 0 and day 3 and maintained on their respective dietary condition (GFD or GRD) (Figure 1H). One week post-colonization, mice were euthanized. Small intestine luminal contents were collected for 16S rRNA gene sequencing, and distal ileum tissue was harvested for histological analysis.

### GF *Streptococcus lutetiensis* colonization experiments

The bacterial strain (*S. lutetiensis*, Cat. No. MS-10669B) was obtained from Creative BioMart. Cultures were revived from a freeze-dried pellet and grown in a custom tomato broth medium at 37°C under aerobic conditions with supplemental carbon dioxide prior to storage in glycerol at −80°C. To prepare the liquid tomato broth medium, the following components were combined in 900  mL of distilled water: 7.5 g yeast extract (Thermo Scientific™, Cat. No. H26769.22), 10 g glucose (Sigma-Aldrich, Cat. No. 67021-1006), 100 mL commercially available tomato juice, 7.5 g peptone (Fisher Scientific™, Cat. No. BP1420-100), 2.0 g monopotassium phosphate (Sigma-Aldrich, Cat. No. P2222-1006), and 5 mL Tween 80 (Research Products International, Cat. No. P20390-0.5). The pH was adjusted to 7.0 prior to autoclaving. For solid medium, 15 g of agar (Fisher Scientific™, Cat. No. BP1423-500) was added to the broth formulation before sterilization. To investigate the diet-dependent effects of *Streptococcus lutetiensis* on host immunity, GF C57BL/6 mice were singly housed individually as described above and placed on either a nutritionally matched GFD or GRD for one week prior to bacterial colonization. Bacterial suspensions from glycerol stocks were plated overnight and then a single colony was grown to log phase (approximately 4 hours.) in sterile media immediately prior to gavage (Supplementary Fig. 4A). Mice were then orally gavaged with 100 μL (approximately 10^6^ CFUs) of log-phase *S. lutetiensis* suspension and maintained on their respective diets for an additional two weeks. Control mice were gavaged with sterile media. Fecal pellets were collected at days 0, 3, 7, and 14 post-colonization for longitudinal assessment of microbial growth and PCR quantification of fecal pellets with strep-specific primers^22^ was utilized to further validate strep colonization. A total of 14 d post-colonization mice were euthanized, and small intestinal (SI) contents were harvested for CFU quantification, distal ileum was collected for histological analysis, and a 15 cm segment of distal SI was processed for flow cytometric analysis of mucosal immune cell populations.

### 16S rRNA gene sequencing

Ileal contents were collected from animals and placed into 2 ml collection tubes containing garnet stones for bead beating following the Powerfecal DNA isolation kit instructions (Qiagen). To remove ileal contents, mice were euthanized and then the ileum was removed with sterile forceps and scissors. The ileal contents were squeezed directly into a collection tube, and DNA extractions were carried out in a biosafety cabinet. Isolated DNA was submitted to the University of Alabama Heflin Center Genomics Core for 250 bp paired-end 16S sequencing on an Illumina MiSeq instrument. Fastq reads were de-multiplexed and forward and reverse primer sequences were trimmed from reads which left a 250 bp product within the V3/V4 region of the bacterial 16S rRNA gene. Demultiplexed fastq files were imported as QIIME 2.0 artifacts for downstream analysis using the Qiime 2.0 pipeline.^23^ ^,^ ^24^ Demultiplexed reads were trimmed based on a quality score cutoff of Q > 15. DADA2 was used for denoising with amplicon sequence variant (ASV) taxonomic calls being made against the GreenGenes database. DADA2 was also used to remove chimeras and low-quality reads. Singletons and reads present in only two or fewer samples were removed. Feature table reads were rarified to even the sampling depth before *β*-diversity analysis. To predict microbial metagenome functional content, the feature table generated by DADA2 was exported from QIIME 2 and imported into the Phylogenetic Investigation of Communities by Reconstruction of Unobserved States (PICRUSt2) pipeline.^25^ ASVs were normalized to 16S rRNA copy numbers, and functional metagenomic predictions were inferred. Pathway abundances and enzymatic functions were annotated using KEGG Orthology (KO) identifiers. To link predicted KOs to metabolic pathways, the KO predictions were processed using the ggpicrust2 pipeline in R. This pipeline was used to convert KO abundances to KEGG pathway-level abundances.

### Flow cytometry

Flow cytometry for immunophenotyping was performed on BD FACSymphony A5SE Cell Analyzer and BD FACSDiscover S8 Cell Sorter. A total of 15 cm of distal small intestine (comprising the entire ileum and half the jejunum) was harvested from mice and immune cells were isolated from the *lamina propria* as previously described.^26^ Approximately 500,000 cells in a volume of 250 μl were pipetted in a 96-well round-bottomed plate. For T-cell immunophenotyping, cells were in incubated for 4 hours in an activation cocktail (Biolegend, cat# 423303) at 37°C with 5% O_2_. Cells were washed once with staining buffer (1XHBSS + 5 mL FBS + 5 mL 0.5 M EDTA). Cells were then incubated with 100 μl of TruStain FcXTM PLUS (Biolegend) at a 1:500 dilution using column buffer to block Fc receptors. After 5 minutes, excess block was removed, and surface marker stains were added. The cell marker stains used for immune cell subsets and are listed with their fluorophores in Supplementary Table T1. Surface stains were diluted 1:500 in column buffer and 100 μl of stain was added per well. Cells were incubated with surface markers for 20 minutes in the dark at room temperature and then washed again with staining buffer. Intracellular and intranuclear stains were diluted 1:50 in intracellular or intranuclear perm-wash buffer. Cells were then incubated with 50 μl of intracellular or intranuclear markers for 20 minutes at room temperature. After this, cells were washed with column buffer and fixed with 4% paraformaldehyde at 1:1 staining buffer:PFA and analyzed. For single stain controls, Slingshot SpectraComp Compensation Beads (cat #SSB-05-B) were stained with antibodies [1:500] and incubated for 20 minutes before being washed with column buffer.

### Statistical analysis

Statistics for all experiments were carried out using GraphPad Prism 9 unless otherwise indicated. Normality was assessed using the Shapiro‒Wilk test. For simple pairwise comparisons of normally distributed data, two-tailed unpaired Student's t-tests were used. For non-normally distributed data, Mann‒Whitney U tests were applied. To assess the main effects of genotype, diet, colonization, and their interactions, two-way ANOVA was used where appropriate. For post hoc multiple comparisons following ANOVA, pairwise comparisons were adjusted using Bonferroni correction. Alpha diversity group comparisons performed in QIIME 2 used Kruskal‒Wallis tests, with pairwise q-values reported where generated by the QIIME 2 alpha-group-significance workflow. Beta diversity group significance was assessed in QIIME 2 using PERMANOVA. Predictor screening was performed using JMP16 software (SAS). Means are shown in figures as measures of central tendency, with individual data points shown to illustrate dispersion. All error bars reflect standard deviation. For experiments pooled across independent cohorts, individual animals are shown as data points unless otherwise indicated. The number of independent experiments contributing to each analysis is provided in the corresponding figure legends. Microsoft Excel was used to generate some graphs, including pie charts, PCoA plots, heatmaps, and *S. lutetiensis* growth curves. All experimental schematics shown in figures were created using BioRender (www.biorender.com).

## Results

### Dietary gluten induces mucosal defects in the small bowel of IgA-deficient mice

Previously, mice deficient in the production of IgA (a different strain than that used in our experiments) were found to develop ileitis driven by enhanced CD4^+^ T-cell responses.^27^ To confirm that a similar phenotype develops in the IgA^−/−^ mouse strain we used in this study, we quantitatively assessed ileal tissues for differences in mucosal architecture in WT and IgA^−/−^ mice independently reared and maintained in our colony on a standard gluten-containing mouse chow (Envigo 2920X). Villus-to-crypt ratio (VCR) scoring was utilized as our primary method for assessing changes to mucosal architecture in response to dietary gluten (Figure 1A). Quantitative assessment revealed that IgA^−/−^ mice develop a reduced VCR ratio which is driven by significant shortening of villi compared to WT mice (Figure 1B and C). Next, SILP immune cells in the distal SI were assessed by flow cytometry and IgA-deficient mice were found to have significantly increased abundances of Th17 cells and reduced NKT cell abundance compared to WT littermates (Figure 1D, Supplementary Fig. 1A). To determine whether this established histologic phenotype remained responsive to dietary intervention, adult IgA^⁻/⁻^ mice maintained on standard gluten-containing chow were switched to a GFD for four weeks. Gluten withdrawal significantly increased ileal VCR in adult IgA^⁻/⁻^ mice, suggesting that villus blunting is at least partially reversible following removal of dietary gluten (Supplementary Fig. 1B). Having identified a gluten-responsive histologic phenotype in IgA^⁻/⁻^ mice, we next used a controlled dietary approach to assess genotype, diet, and genotype x diet interactions. WT or IgA^−/−^ mice were weaned onto nutritionally matched GFD or GRD for four weeks, after which ileal histological assessments were performed (Figure 1E). VCR scoring showed a significant genotype × diet interaction in that IgA^−/−^ mice maintained on a GRD had significantly reduced VCR and shorter villi than IgA^−/−^ littermates maintained on a nutritionally matched GFD. The presence or absence of dietary gluten did not differentially influence mucosal architecture in the SI of WT mice (Figure 1F and G). Although gluten-related disorders in humans are often proximal, we did not observe a duodenal phenotype in GRD-fed IgA^⁻/⁻^ mice (Supplementary Fig. 1C), consistent with our previous findings that antibody deficiency-associated enteropathy is most evident in the ileum^20^ ^,^ ^21^ ^,^ ^26^ ^,^ ^28^; therefore, subsequent histologic, immunologic, and microbiome analyzes focused on the ileum. Sex-stratified analysis of ileal VCR showed that this gluten-dependent reduction in mucosal architecture was observed across male and female IgA^⁻/⁻^ mice, with no evidence that sex was the primary driver of the phenotype (Supplementary Fig. 1D).

To test whether the interaction between gut microbes and dietary gluten was sufficient to induce villus blunting, we utilized germfree mice, which are known to have reduced production of endogenous IgA.^29–31^ For this experiment, germfree C57BL/6 mice were first maintained on nutritionally matched GFD or GRD for one week, followed by one week of colonization with small intestinal microbiota isolated from SPF WT or IgA^⁻/⁻^ donor mice maintained on standard gluten-containing chow (Figure 1H). This approach allowed us to assess whether transferred SI microbial communities could interact with dietary gluten to rapidly alter mucosal architecture. Results from this experiment demonstrate a significant colonization × diet interaction in that microbial colonization in the presence of dietary gluten led to villus blunting regardless of donor genotype (Figure 1I). 16S analysis of SI contents from colonized GF mice showed microbial shifts after one week of colonization. Specifically, we report a significant (2-fold) increase in observed species on a GRD compared with a GFD in both WT and IgA SIM colonized mice (Supplementary Fig. 1E). Additionally, beta diversity analysis revealed significant compositional shifts in GF mice colonized with both WT and IgA^−/−^ SIM (Supplementary Figs. 1F, 1G). Together, these data indicate that dietary gluten promotes microbial expansion and compositional remodeling in colonized GF mice, and that villus blunting is driven by the interaction between dietary gluten and transferred SI microbial communities rather than inherent pathogenicity of the IgA^⁻/⁻^ microbiota.

### IgA deficiency results in diet-dependent enhancement of T_H_17 responses and genotype-dependent defects in natural killer-like T (NKT) cell homeostasis

**Figure 2.**
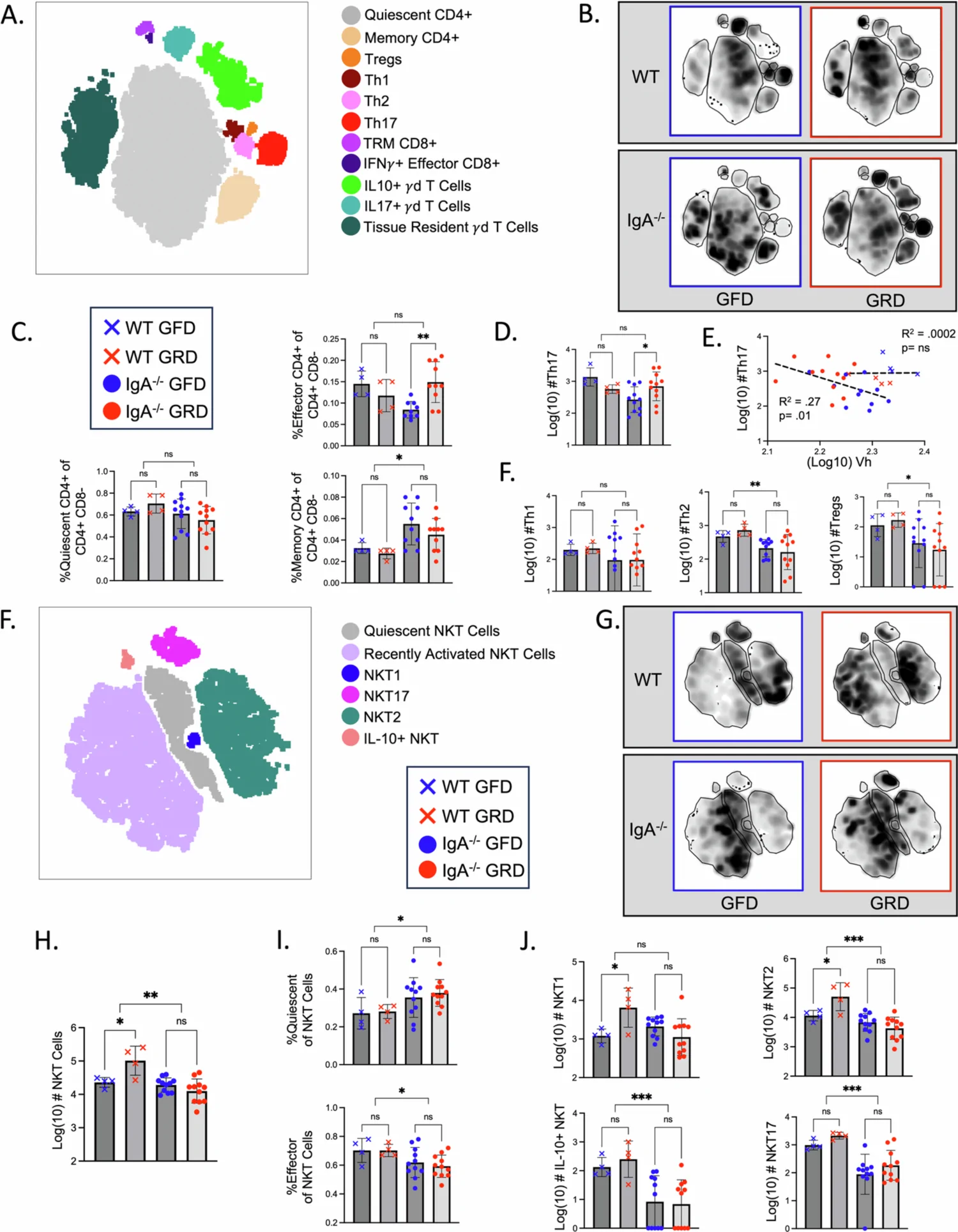
IgA deficiency results in a diet-dependent enhancement of the Th17 response and genotype-dependent defects in iNKT cell homeostasis. (A) Representative tSNE plot of SI resident T (CD45+ , CD3+, NK1.1−) cell subsets analyzed by high-dimensional flow cytometry (B) Representative tSNE density plots of T-cell subsets in WT (GFD *n* = 4, GRD *n* = 4) and IgA^−/−^ (GFD *n* = 11, GRD *n* = 11) on a GFD or GRD. (C) Relative abundance of effector, memory and naïve CD4+  (CD3+, NK1.1−, TCR β +, CD4+) T-cell subsets. (D) Absolute abundance of T helper subsets by intracellular cytokine profiling (E) Correlation between Vh in WT and IgA^−/−^ mice on a GFD or GRD. (F) Representative tSNE plot of SI resident NKT (CD3+ , NK1.1+, TCR β +) cell subsets analyzed by high-dimensional flow cytometry. (G) Representative tSNE density plots of NKT-cell subsets in WT and IgA^−/−^ on a GFD or GRD. (H) Absolute abundance of NKT cells (CD3+ NK1.1+ TCR β +) and relative abundance of quiescent (CD44-) and effector (CD44+) NKT (CD3+ , NK1.1+ , TCR β +) cell subsets. (I) Absolute abundance of effector NKT subsets by intracellular cytokine profiling and correlation with Vh in WT and IgA^−/−^ mice on a GFD or GRD. Data Two-way ANOVA showing “genotype”, “diet” or “genotype × diet” interactions with ad hoc comparisons to show significant interactions; ns = not significant, * = *p* < 0.05, *** = *p* < 0.001, **** = *p* < 0.0001. All data are pooled across two replicate experiments.

To investigate the immune phenotype of IgA-deficient mice in the presence or absence of dietary gluten, WT and IgA^−/−^ mice were singly housedand fed either a GFD or a nutritionally matched GRD for four weeks. Following the diet period, *lamina propria* immune cells were isolated from the distal ileum and analyzed using high-dimensional flow cytometry (Figure 2A; Supplementary Figs. 2A, 2E). T-cell phenotyping revealed significant “genotype × diet” interaction effects on the abundance of CD4^+^ T helper cell subsets (Figure 2B; Supplementary Figs. 2B-2D). Specifically, GRD-fed IgA^−/−^ mice exhibited a significant increase in the frequency of effector (CD4^+^CD69^+^CD44^+^) CD4^+^ T cells (Figure 2C). Intracellular cytokine profiling within the CD4^+^ effector T-cell population demonstrated that the increase in effector CD4^+^ cells was driven by an increased absolute abundance of effector T_h_17 (CD44^+^CD69^+^ IL-17^+^IFNγ^−^) cells (Figure 2D). The abundance of T_h_1, T_h_2, and regulatory T cells (Tregs) were not affected by dietary gluten, but IgA^−/−^ mice were observed to have fewer Tregs (IFNγ^−^IL-17^−^IL-4^−^IL-10^+^) and Th2 (IFNγ^−^IL-17^−^IL-4^+^IL-10^−^) cells than WT mice regardless of diet (Figure 2D). Dietary gluten did not alter T-cell phenotypes in WT mice (Figure 2B–D; Supplementary Figs. 2B–2D). The frequency of T_h_17 cells was negatively correlated with villus length; specifically in IgA^−/−^ mice (Figure 2E).

sIgAD also resulted in alterations in NKT cell responses (Figure 2F and G; Supplementary Fig. 2F). Specifically, IgA^−/−^ mice had fewer NKT cells in total than WT mice (Figure 2H) and a significantly higher proportion of them were not activated (i.e. quiescent) (Figure 2I). Consistent with reduced NKT cell activation, IgA^−/−^ mice had fewer NKT17, NKT2, and IL-10+ NKT cells in the SILP compared with WT mice (Figure 2J). Interestingly, we observed a significant “Diet × Genotype” interaction in NKT cell activation in WT mice (Supplementary Fig. 2F). Specifically, GRD-fed WT mice had increased abundance of total NKT cells compared with GFD-fed WT mice (Figure 2H). This was driven primarily by an expansion of NKT1 and NKT2 cells (Figure 2J).

### IgA-deficiency makes mice sensitive to gluten-induced shifts in microbial composition and function

**Figure 3.**
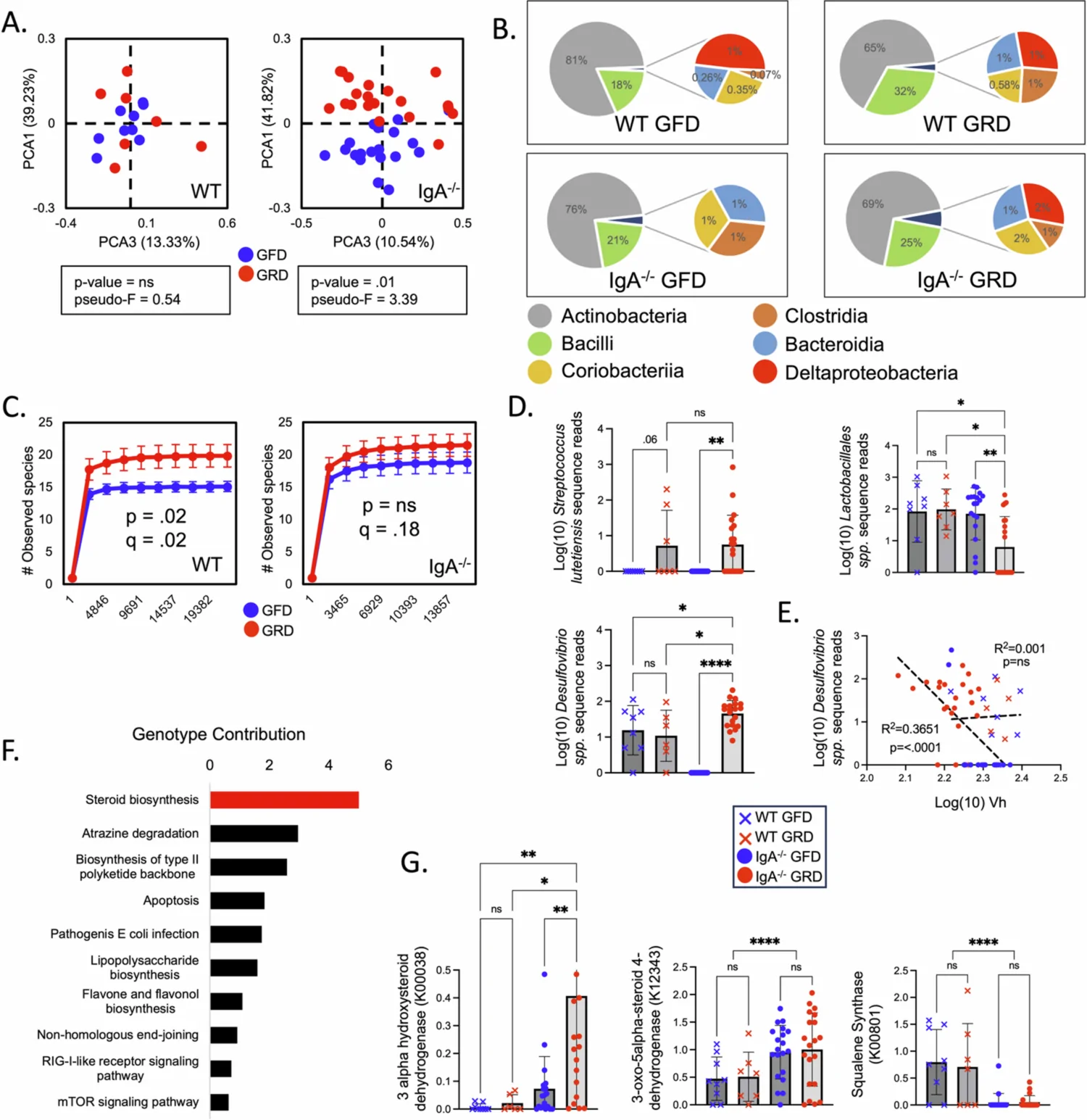
Mucosal IgA maintains microbial composition in response to dietary gluten (A) Bray‒Curtis distance matrix showing microbial beta diversity of ileal contents in WT (*n* = 16) and IgA^−/−^ (*n* = 39) mice on a GFD or GRD. (B) Representative pie charts showing class-level microbial composition between WT and IgA^−/−^ on GFD and GRD. (C) Observed species in WT and IgA mice on a GFD and GRD. (D) Dietary gluten leads to outgrowth *Streptococcus spp.* in both WT and IgA^−/−^ mice and outgrowth of *Desulfovibrio spp.* and loss of *Lactobacillales spp.* in IgA^−/−^ but not WT mice. (E) Linear regression between *Desulfovibrio spp.* and villus height in WT and IgA^−/−^ mice on a GFD or GRD. (F) Bar plot depicts the “contribution” of predicted microbial pathway (from PICRUSt2 analysis) to group separation as determined by predictor screening in JMP. The steroid biosynthesis pathway (red) shows the highest contribution to predicting genotype. Values above bars represent JMP LogWorth scores, calculated as −log10 (*p*-value) (G) Predicted enzyme abundances of three significant enzymes from the steroid biosynthesis pathway between all treatment groups. Two-way ANOVA showing “genotype”, “diet” or “genotype × diet” interactions with ad hoc comparisons to show significant interactions; ns = not significant, * = *p* < 0.05, *** = *p* < 0.001, **** = *p* < 0.0001. PERMANOVA pseudo-F reflects between-group separation. Data are pooled across three replicate experiments.

To assess the influence of dietary gluten on microbial ecology in the SI, DNA was extracted from small intestine luminal contents of WT and IgA^⁻/⁻^ mice that were singly housed on either a GFD or GRD for four weeks. Results from our 16S analysis revealed a significant genotype × diet interaction in microbial composition. Surprisingly, Bray‒Curtis analysis showed that four weeks on a GFD or GRD significantly altered ileal microbial composition in IgA^⁻/⁻^ mice, but not in WT mice (Figure 3A). We further assessed all four groups together using unweighted UniFrac to compare phylogenetic community structure across all four treatment groups. This analysis similarly showed a significant diet-associated shift within IgA^⁻/⁻^ mice and no significant diet-dependent separation within WT mice. A genotype-associated separation between WT and IgA^−/−^ mice was most apparent under GFD conditions (Supplementary Figs. 3A, 3B). Sex-stratified analysis did not identify sex as a major driver of ileal microbial community separation within genotype and diet groups (Supplementary Fig. 3C). The observed compositional shift in IgA^⁻/⁻^ mice was not due to a broad loss or gain of bacterial species (Figure 3C), but rather altered abundance of existing species. Consistent with this, taxonomic overlap analysis showed substantial overlap in detected taxa between GFD and GRD conditions within each genotype, indicating that diet-associated differences primarily reflected shifts in abundance rather than complete gain or loss (Supplementary Fig. 3D). Genotype comparisons within each diet showed greater divergence in detected taxa between WT and IgA^⁻/⁻^ mice regardless of diet, with more unique species in IgA^⁻/⁻^ mice (Supplementary Fig. 3E).

Taxonomic analysis revealed a significant genotype × diet interaction, with GRD-fed IgA⁻/⁻ mice exhibiting significant outgrowth of *δ*-Proteobacteria compared with WT (GFD and GRD) and IgA⁻/⁻ GFD groups (Figure 3B), driven by a single unidentified Desulfovibrio taxon. GRD-fed IgA⁻/⁻ mice also had a significant reduction in Lactobacillales compared with GFD-fed IgA⁻/⁻ mice and WT (GFD and GRD) mice (Figure 3B and D). Notably, a diet effect was also observed for *Streptococcus lutetiensis*, which expanded in both WT and IgA⁻/⁻ mice on a GRD diet. The corresponding level-7 abundance heatmap further summarized these genotype- and diet-associated taxonomic patterns across groups (Supplementary Fig. 3F). Importantly, Desulfovibrio spp. abundance was significantly negatively correlated with villus length in IgA⁻/⁻ mice but not WT mice (Figure 3E).

To identify potential microbial functional pathways predictive of genotype- and diet-associated effects, we used PICRUSt2 to infer KEGG pathway abundances,^25^ followed by pathway enrichment analysis using the ggpicrust2 R plugin.^32^ Pathway enrichment analysis revealed that the steroid biosynthesis pathway (KO00100) was the strongest predictor distinguishing microbiota from IgA^−/−^ vs. WT mice (Figure 3F). Three enzymes were identified as significantly altered in the dataset: 3-alpha hydroxysteroid dehydrogenase (K00038), 3-oxo-5alpha-steroid 4-dehydrogenase (K12343), and squalene synthase (K00801). Notably, 3-alpha hydroxysteroid dehydrogenase, critical for steroid transformations especially in bacterial bile acid metabolism, showed a genotype × diet interaction and was enriched in IgA^−/−^ on a GRD compared to all other groups (Figure 3G). 3-oxo-5alpha-steroid 4-dehydrogenase, another key enzyme in microbial bile acid metabolism, was influenced by genotype only and reduced in WT mice and increased in IgA^−/−^ deficient mice. (Figure 3G). Squalene synthase, an enzyme in the microbial sterol or hopanoid synthesis pathway, also showed a genotype effect and was increased in WT mice compared to IgA^−/−^ regardless of diet. (Figure 3G).

### Dietary gluten promotes SI-specific T-cell expansion and mucosal remodeling in response to *Streptococcus lutetiensis* colonization in germ-free mice

**Figure 4.**
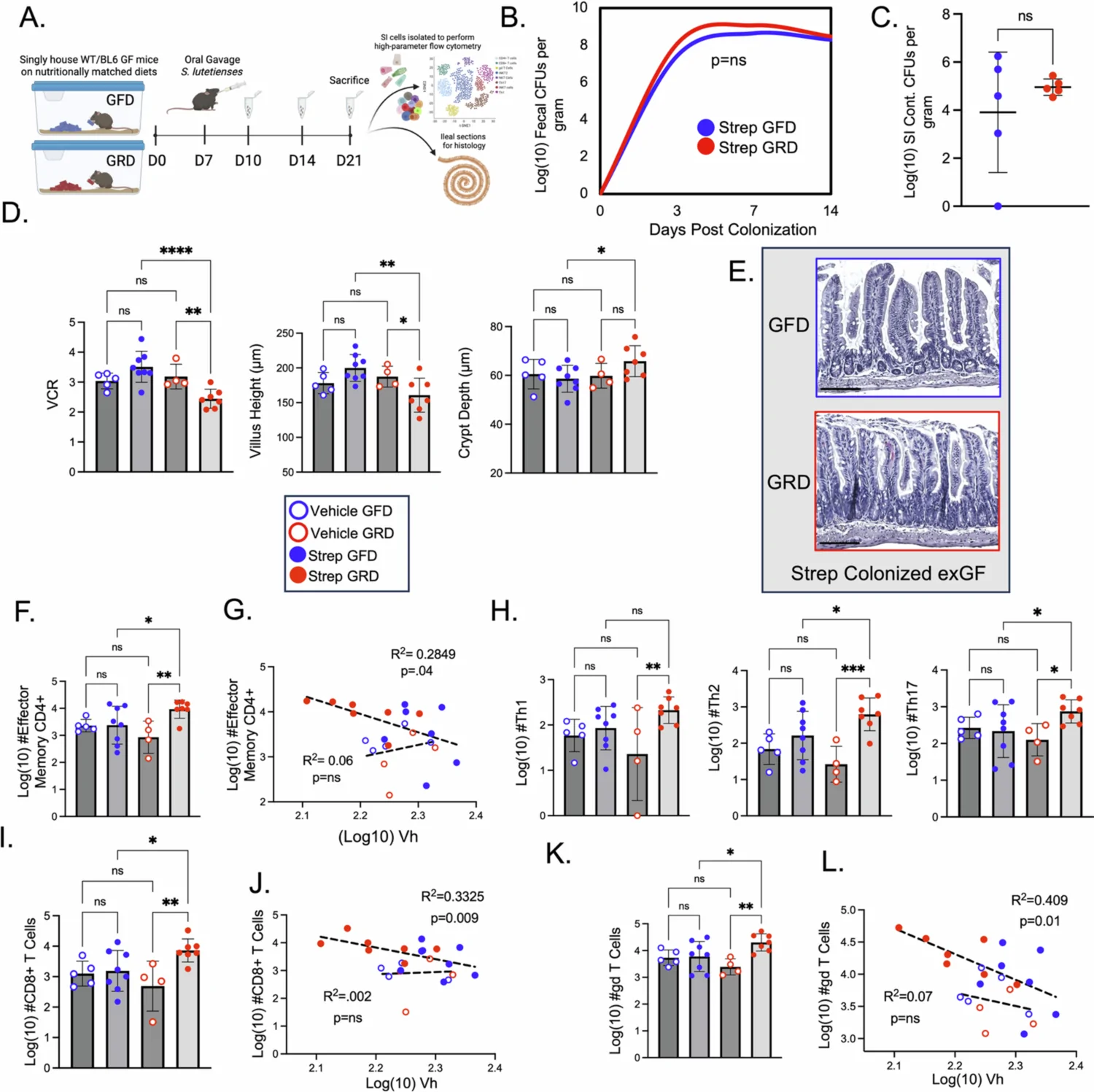
Dietary gluten promotes SI-specific T-cell expansion and mucosal remodeling in response to *Streptococcus lutetiensis* colonization in germ-free mice. (A) Schematic of *S. lutetienses* colonization in C57BL/6 germ-free mice on a GFD or GRD. (B) Colonization growth curve day 0–14 post-colonization based on fecal CFU counts normalized by pellet weight. (C) Day 14 CFU counts from SI contents at sacrifice. (D) VCR scoring of distal ileum in colonized and ctrl mice on a GFD or GRD. (E) Representative images of distal ileum of colonized mice on a GFD or GRD. (F) Absolute abundance of effector memory CD4+ T Cells (TCR β + CD4+ CD44+− CD69+). (G) Linear regression between effector memory CD4+ and villus height of colonized mice. (H) Absolute abundance of Effector memory CD4+  T helper subsets by intracellular cytokine profiling. (I) Absolute abundance of CD8+ T Cells (TCR β + CD8+ CD4−) (J) Linear regression between CD8+ T cells and villus height in colonized mice. (K) Absolute abundance of γδ T Cells (TCR γδ + TCR β -). (L) Linear regression between γδ T cells and villus height in colonized mice. Two-way ANOVA showing “colonization”, “diet” or “colonization × diet” interactions with ad hoc comparisons to show significant interactions; ns = not significant, * = *p* < 0.05, *** = *p* < 0.001, **** = *p* < 0.0001. Data are pooled across 2 replicate experiments.

To test whether a single species of bacteria was able to interact with dietary gluten to drive villus atrophy, we mono-colonized GF C57BL/6 mice with *Streptococcus lutetiensis*, a pathobiont that our lab has consistently linked to gluten-induced atrophy in other mouse models of antibody deficiency (CD19^−/−^ and J_H_^−/−^ mice),^20^ ^,^ ^21^ as well as in our current study. GF mice were maintained on either a GFD or nutritionally matched GRD for one week prior to colonization, then gavaged with log-phase *S. lutetiensis* and subsequently maintained on the same diet for two additional weeks (Figure 4A). PCR identification in fecal pellets validates strep colonization (Supplementary Fig. 4B). Fecal and SI CFU counts confirmed stable engraftment across both dietary groups, with no significant differences in bacterial burden between them at any time point (Figure 4B and C). These results indicate that dietary gluten does not influence *S. lutetiensis* colonization efficiency in the GF setting.

Despite similar levels of colonization, GRD-fed mice developed significantly reduced villus-to-crypt ratios driven by villus blunting compared to GFD-fed counterparts and uncolonized controls (Figure 4D and E), consistent with mucosal remodeling. Flow cytometric analysis (Supplementary Fig. 4C) revealed a significant diet × colonization effect driven by an expansion of effector memory CD4⁺ T cells (TCRβ⁺CD4⁺CD44⁺CD69⁺) in the SI of colonized GRD-fed mice (Figure 4F), and the abundance of this population negatively correlated with villus height in colonized mice (Figure 4G). Total CD45⁺ leukocyte frequencies showed no significant diet, colonization, or diet × colonization interaction effects (Supplementary Fig. 4D), indicating that the response was not due to generalized immune infiltration. However, CD3ε⁺ T cells were significantly increased in GRD-fed colonized mice compared to all other groups, including GFD-fed colonized mice (Supplementary Fig. 4D). Intracellular cytokine profiling showed that this T-cell response was skewed toward Th1 and Th17 subsets (Figure 4H), both of which are associated with barrier-disruptive inflammation. CD8⁺ T cells and TCRγδ^+^ T cells were also increased in the SI of GRD-fed mice (Figure 4I–K; Supplementary Fig. 4E), and both cell types were inversely correlated with villus height in colonized mice (Figure 4J–L), further supporting a link between gluten-sensitive immune activation and epithelial remodeling. Together, these data indicate that *S. lutetiensis* is not intrinsically sufficient to induce villus blunting in germ-free mice but instead promotes SI T-cell expansion and mucosal remodeling in a gluten-dependent manner.

## Discussion

The functional role of sIgA in maintaining gut microbial homeostasis and preventing dysbiosis is well established, although its importance in steady-state conditions is often underappreciated as the impact of IgA deficiency is masked by compensatory levels of IgG and IgM.^5^ ^,^ ^33^ sIgA plays a central role in preventing the overgrowth of pathobionts and regulating the spatial organization of the gut microbiota and limiting their access to the intestinal epithelium. In its absence, microbial imbalances (i.e. dysbiosis) can arise, leading to impaired barrier function, increased mucosal exposure to antigens, and enhanced inflammatory immune responses.^5^ ^,^ ^34^ Recent studies, including by Nagaishi et al., have shown that IgA^−/−^ mice develop ileitis due to the expansion of colonic-type bacteria into the small intestine and subsequent activation of T_h_17-driven inflammation.^27^ In previous studies, we demonstrated that B-cell-deficient CD19^−/−^ and J_H_^−/−^ mouse models of humoral immunodeficiency develop gluten-sensitive ileitis driven by dysbiosis and expansion of inflammatory T cells, especially T_h_17 cells.^21^ ^,^ ^28^ Here, we extend these observations to IgA^−/−^ mice which exhibited gluten-sensitive, SI-specific enteropathy characterized by villus blunting, increased T_h_17 responses, and the outgrowth of *Desulfovibrio* spp., all of which were alleviated by a gluten-free diet, highlighting the interplay between mucosal antibody deficiency, diet, and microbial composition in driving enteropathy in the small bowel.

Emerging evidence suggests that gluten not only acts as an immunogenic trigger in susceptible hosts but may also contribute to dysbiosis by functioning as a pro-inflammatory prebiotic that fuels the growth of pathobionts. For example, in an earlier study, Caminero et al. demonstrated that 144 bacterial strains cultured from human fecal samples could metabolize dietary gluten, underscoring gluten's potential to shape microbial communities.^19^ Additionally, another study found a loss of diversity in gluten-degrading bacteria in the stool of ulcerative colitis (UC) patients compared to healthy controls, with an enrichment of gluten-degrading *Enterococcus* spp. in UC patients, suggesting that shifts in microbial populations due to the ingestion of dietary gluten may promote inflammation in disease states.^35^ Some of these bacteria, including *Streptococcus* and *Enterococcus* species, are also known to encode microbial transglutaminases (mTGs), which can cross-link gliadin peptides and potentially enhance their immunogenicity. A wealth of data supports that the presentation of immunogenic gluten peptides by patients possessing specific HLA susceptibility alleles drives T-cell-mediated enteropathy in celiac disease^36^; the most commonly diagnosed form of gluten sensitivity in humans. The remainder of our discussion will focus on the consequences of dietary gluten's role as a prebiotic and alternative pathways through which it may drive villus atrophy. Some of these effects may explain why gluten-sensitive enteropathies also develop in patients that do not possess known HLA susceptibility alleles.

Our results strongly implicate disruptions to the microbiota in driving gluten sensitivity in sIgAD. *Streptococcus* species are prominent members of the small intestinal microbiota and have long been implicated in shaping mucosal immunity and disease susceptibility. Several members of the *Streptococcus* group—including *S. lutetiensis*, *S. sanguinis*, and *S. intermedius* have been isolated from both human feces and inflamed tissue in celiac patients.^18^ ^,^ ^19^ Our lab has also previously demonstrated that a *Streptococcus* species (*S. lutetiensis*) was outgrown in both CD19^−/−^ and J_H_^−/−^ mice on a GRD, potentially contributing to enteropathy.^20^ ^,^ ^21^ While some *Streptococcus* species are known to encode mTGs, it remains unknown whether members of the *S. bovis* group—including *S. lutetiensis*—express functional mTGs *in vivo*. In livestock, overgrowth of *S. bovis* on wheat-based diets leads to lactic acidosis, mucosal degradation, and impaired nutrient absorption, supporting the plausibility of a diet–microbe interaction in pathogenesis. In the current model, we found *S. lutetiensis* to be differentially outgrown in the presence of gluten in both WT and IgA^−/−^ mice. To test its causal role in villus atrophy, we mono-colonized germ-free mice with *S. lutetiensis* and observed that, in the presence of dietary gluten, colonization was sufficient to induce villus blunting and a marked expansion of inflammatory T-cell subsets, including Th1, Th2, Th17, CD8⁺, and TCRγδ^+^ T cells. These effects were absent in GFD-fed mice, indicating that *S. lutetiensis*–driven inflammation is gluten-dependent and not simply a result of colonization. Importantly, *S. lutetiensis* did not overgrow in GRD-fed animals, suggesting that gluten may instead alter its function in the small intestine to drive inflammation.

*Desulfovibrio* spp. are also significantly enriched in IgA^−/−^ mice on a GRD, and their abundance significantly correlates with villus blunting. *Desulfovibrio* spp. thrive in anaerobic conditions and produce hydrogen sulfide through sulfate reduction, a process that, when dysregulated, can lead to epithelial damage by impairing tight junctions and promoting mucosal inflammation.^37–41^ This overgrowth likely exacerbates inflammation through H₂S-mediated epithelial damage and the recruitment of proinflammatory immune cells. While *Desulfovibrio* spp. have not been found to directly degrade gluten, they may benefit from cross-feeding interactions within the gut microbiota, utilizing byproducts of gluten fermentation as substrates.^42^ Additionally, dietary gluten may increase mucus production and provide a nutrient-rich environment that further supports their expansion. The exclusion of dietary gluten in IgA^−/−^ mice effectively eliminated *Desulfovibrio* abundance and its potentially harmful effects on intestinal barrier function.

Predictive functional analysis of 16S data (PICRUSt) highlights a significant influence of sIgAD and dietary gluten on microbial steroid biosynthesis (ko00100), underscoring how mucosal immunity modulates microbial metabolic pathways involved in lipid homeostasis and bile acid metabolism. One key finding in our study is the increased abundance of the gene encoding the enzyme 3-alpha hydroxysteroid dehydrogenase (3α-HSDH) in IgA^−/−^ mice on a GRD, suggesting a microbial-driven disruption in bile acid pools. While 3α-HSDH is essential for the synthesis of secondary bile acids,^43^ its overactivity may lead to excessive oxidation of secondary bile acids like LCA and DCA, resulting in the accumulation of 3-oxo intermediates that are not efficiently converted into beneficial epimers like isoDCA and isoLCA.^44^ ^,^ ^45^ This imbalance diminishes the production of anti-inflammatory bile acids, impairing their ability to promote Treg differentiation and suppress Th17 responses.^46^ Compounding this effect, increased abundance of the gene encoding the enzyme 3-oxo-5α-steroid 4-dehydrogenase (K12343) in IgA^−/−^ mice highlights an additional putative route for generating steroid intermediates that fail to support immune regulation. In support of this, previous studies in our lab have shown that B-cell-insufficient CD19^−/−^ mice present with gluten-sensitive enteropathy as well as defects in bacterial bile acid metabolism.^20^ ^,^ ^28^

Further analysis of functional differences revealed that microbial squalene synthase (K00801), an enzyme catalyzing the conversion of farnesyl diphosphate to squalene, a precursor for bacterial sterol and hopanoids,^47^ ^,^ ^48^ was significantly reduced in IgA^−/−^ mice. Several studies have identified health benefits related to squalene supplementation in the alteration of gut and serum lipoprotein profiles as well as reduction in oxidative stress in mice.^49^ Additionally, dietary squalene was found to induce intestinal tissue repair in a porcine model, reducing the abundance of harmful bacteria, including *Desulfovibrio.* ^50^ Microbial disruptions in lipid metabolism and steroid biosynthesis may be key mechanisms through which sIgAD promotes gluten-induced inflammation and dysbiosis. The functional shifts could explain the improved immune and histological outcomes observed in WT mice. By promoting distinct taxonomic and functional profiles, mucosal IgA may precondition the microbial environment to support effective bacterial bile acid metabolism, lipid metabolism, and lipid antigen presentation, thereby influencing NKT cell activation and mitigating Th17-driven inflammation. NKT cells play a key role in maintaining intestinal barrier integrity and limiting inflammatory responses.^26^ ^,^ ^51^ These lymphocytes can be activated by lipid or protein antigens and rapidly produce cytokines to regulate immune homeostasis.^51^ Reduced NKT cell populations have been observed in patients with inflammatory bowel disease (IBD), CeD, and other gut disorders, suggesting that their deficiency contributes to disease progression.^52^ ^,^ ^53^ In a recent study by our group, NKT cell quiescence was a major driver of immune dysregulation associated with SI enteropathy in a series of antibody-deficient mouse models, including sIgAD.^26^ Notably, several human studies have also reported NKT cell deficiency in CVID patients.^54–57^ In the current study, we again report that IgA^−/−^ mice exhibit increased NKT cell quiescence and a significant reduction in effector NKT cell subsets compared to WT mice, regardless of diet. Notably, dietary gluten induced robust NKT cell activation only in WT^26^ ^,^ ^28^ mice. This suggests that NKT cell responses to microbial and dietary cues may serve as a protective mechanism that is impaired in the absence of sIgA. This impairment may contribute to a skewing toward inflammatory CD4⁺ T-cell responses in response to microbial shifts.

Several limitations of this study also establish important future directions. Although gluten-associated pathology in humans is classically most prominent in the proximal small intestine, the phenotype in this model was ileum-predominant. This regional difference should be considered when interpreting the translational relevance of the model; however, ileal inflammation is a common feature of many murine models of dysbiosis-driven enteropathy, including antibody-deficiency and Crohn's-like ileitis models.^27^ ^,^ ^28^ ^,^ ^58^ ^,^ ^59^ The distal SI may be particularly vulnerable to microbial imbalance because of its proximity to the colon and susceptibility to small intestinal bacterial overgrowth-like expansion of colonic-type bacteria.^60^ Additionally, in humans, duodenal biopsy is the diagnostic gold standard for gluten-associated enteropathy,^61^ whereas ileal sampling is less commonly performed; therefore, the contribution of ileitis or distal SI immune responses in gluten-sensitive patients remains understudied. Future studies should define the mechanisms that make the distal SI particularly sensitive to sIgAD, dietary gluten, and microbiota-dependent inflammation. Most dietary experiments in this study placed mice on GFD or GRD at weaning and therefore primarily address how dietary gluten shapes the progression of chronic mucosal inflammation in the setting of sIgAD. However, we showed that adult IgA^⁻/⁻^ mice maintained on standard gluten-containing chow showed improved ileal histopathology after four weeks on a GFD, suggesting that established gluten-associated mucosal damage is at least partially reversible. Future longitudinal studies will be needed to define how gluten withdrawal reshapes the microbiota, microbial metabolism, and mucosal immune responses during recovery. Similarly, short-term SIM-transfer provides a useful model for testing whether transferred SI microbial communities can rapidly interact with dietary gluten to induce mucosal remodeling, but it does not define long-term microbial engraftment, community succession, or the stability of these responses over time. Finally, *S. lutetiensis* mono-colonization demonstrates gluten-dependent pathogenic activity, but future studies will be needed to identify the specific microbial and host pathways that mediate T-cell activation and mucosal remodeling and whether these effects are reversible.

Our findings suggest that sIgAD amplifies gluten sensitivity through dysregulated host–microbiota interactions, promoting proinflammatory T-cell responses and epithelial damage. The expansion of *Streptococcus lutetiensis* in GRD-fed IgA^⁻/⁻^ and WT mice—coupled with its ability to induce villus blunting and T-cell activation in germ-free hosts on a GRD—identifies this species as a gluten-sensitive pathobiont. Additionally, the reduction of *Desulfovibrio* spp. and restoration of *Lactobacillales* under gluten-free conditions in IgA^−/−^ mice highlights the importance of diet-microbiota interactions in regulating mucosal immune responses. sIgAD also fosters microbial-driven metabolic disruptions, including altered bile acid and lipid metabolism, which may exacerbate microbiota-driven inflammation via natural killer T (NKT) cell quiescence and enhanced Th17 responses. Collectively, IgA^⁻/⁻^ mice and germfree models provide a powerful tool for investigating microbiota-dependent mechanisms of gluten sensitivity and reveal how diet, immunity, and microbial metabolism converge to shape disease susceptibility.

## Supplementary Material

Supplementary_Figure_3.jpg Supplementary_Figure_3.jpg

Supplementary_Figure_1.jpg Supplementary_Figure_1.jpg

Supplementary_Figure_2.jpg Supplementary_Figure_2.jpg

Supplementary_Figure_4.jpg Supplementary_Figure_4.jpg

Supplementary Tables.docx Supplementary Tables.docx

## Data Availability

All experimental data sets have been made available through the Dryad Data Repository. All 16S rRNA sequences have been made available upon publication through the NCBI SRA repository (PRJNA1356522).
