# Supplementary material for: IgA deficiency reveals a microbiota-dependent pathway to gluten sensitivity
Source: Gut Microbes. 2026 Sep 18;18(1):2724175. doi: 10.1080/19490976.2026.2724175 (PMC13596903; doi:10.1080/19490976.2026.2724175)
Supplement: Supplementary file 1 [file KGMI_A_2724175_SM3015.docx]

**Supplementary Table 1:**

**Supplementary Table 2:**
